# Psychiatric nurses’ experiences implementing a model for constructive group therapy in mood disorders

**DOI:** 10.4102/curationis.v47i1.2577

**Published:** 2024-09-25

**Authors:** Hester M.P. Visagie, Annie Temane, Marie Poggenpoel

**Affiliations:** 1 Department of Nursing, Faculty of Health Sciences, University of Johannesburg, Johannesburg, South Africa

**Keywords:** constructive group therapy, facilitate, implementation, model, mood disorders, psychiatric nurses

## Abstract

**Background:**

In South Africa, various treatment modalities from abroad have been implemented to treat patients with mood disorders. This article is based on a South African model that has been developed, implemented and evaluated for psychiatric nurses to use in facilitating constructive group therapy for patients with mood disorders.

**Objectives:**

This study aimed to describe psychiatric nurses’ experiences in implementation of a model to facilitate constructive group therapy for patients with mood disorders.

**Method:**

A qualitative, exploratory, descriptive and contextual research design was used for this study. Participants were psychiatric nurses working in an inpatient unit for patients with mood disorders in a public psychiatric hospital.

**Results:**

The study revealed that psychiatric nurses experienced the model as a supportive tool to facilitate constructive interaction among patients with mood disorders. The model was beneficial in creating a safe space for patients to share and deal with their challenges, promoting optimal functioning outside the hospital setting. The model’s implementation also fostered improvement in psychiatric nurses’ personal and professional skills.

**Conclusion:**

The model emphasised psychiatric nurses’ importance in treating patients with mood disorders and ensuring positive patient experiences and outcomes.

**Contribution:**

This study contributes to the theory of clinical psychiatric nursing practice and the empowerment of psychiatric nurses, creating self-awareness related to working with patients with mood disorders.

## Introduction

Mental disorders continue to pose a significant challenge worldwide. In South Africa, the status quo is no different, especially in the public health sector, where resources and access to care are limited (Docrat et al. 2019:717; Santomauro 2021:1710; Yearwood & Hines-Martin 2017:3). Throughout the world, health systems are struggling to adequately expand and tailor treatment modalities according to the needs of people with mental health disorders. Mood disorders constitute a large proportion of this global challenge; in fact, depressive disorders are rated as having the highest prevalence rate (Kaltenboeck & Harmer 2018:1; Sadock, Sadock & Ruiz 2015:1400; WHO 2023:n.p.). When considering the associated risk of suicide in patients experiencing depression, the impact of mental and mood disorders becomes an even more alarming concern. Approximately 900 000 people commit suicide every year, ranking suicide as the second-most common cause of death among young people (Sadock et al. 2015:1400). Suicide also accounts for up to 15.4% of deaths per 100 000 of the global population (WHO 2018:n.p.).

Mood disorders are pathological disorders characterised by prominent affective and cognitive disturbances and impairment in psychomotoric and hypothalamic functions (Moini, Koenitzer & LoGalbo 2021:95; Videbeck 2022:285). These patients experience severe emotional distress and struggle, for example, with concentration, memory impairment and an inability to make decisions (Hove et al. 2023:379). Critical thinking skills may also be hampered. Furthermore, recurrent thoughts about death and suicide may be present, resulting in emotional turmoil and feelings of guilt (Ollivier et al. 2022:n.p.). According to the DSM of Mental Health Disorders (American Psychiatric Association 2013:163–164, 167), mood disorders also have a detrimental impact on the individual’s occupational, social and other important areas of functioning. The DSM-5 diagnostic criteria state that symptoms should not be present because of substance use or other medical conditions for the illness to be classified as a mood disorder. Furthermore, another mental illness should not better explain the condition, and no episodes of hypomania or mania should be present (American Psychiatric Association 2013:163–164).

Patients affected by mood disorders often feel overwhelmed, helpless and hopeless (Visagie, Poggenpoel & Myburgh 2020:4) and resort to dysfunctional coping mechanisms and related behavioural patterns like self-harm, substance abuse and suicidal behaviour. These patients’ thought patterns revolve around negative themes, and they often ruminate on these themes, exacerbating already existing emotional distress (Heissel et al. 2023:395).

In South Africa, various treatment modalities from abroad have been implemented to treat patients with mood disorders. Literature ultimately emphasises the effectiveness of psychotherapeutic interventions in the treatment of patients with mood disorders (Gaskell et al. 2022:54; Medina et al. 2022:372; Novick & Swartz 2019:245). Group therapy is therefore an effective treatment modality in treating this population (Rosendahl et al. 2021:56–57), and psychiatric nurses are well placed to offer group therapy for patients with mood disorders (Joseph, Plummer & Cross 2022:697). However, this is rarely the case; in fact, little is known about group therapy facilitated by psychiatric nurses in South Africa.

Alzahrani (2023:45) concurs, stating that irrespective of the effectiveness of psychotherapeutic interventions in treating patients with mental disorders, psychiatric nurses’ role in this type of treatment remains limited. A pervasive view that psychiatric nurses’ roles are limited to the administration of medication, assisting patients with daily activities of living, maintaining ward routines and engaging in administrative functions remains. Hurley et al. (2022:797) align themselves with the aforementioned statement, reflecting that few developers of treatment programmes, if any, acknowledge psychiatric nurses’ potential or their collaborative involvement in the implementation of psychotherapeutic treatment programmes for patients with mental disorders. According to McAllister et al. (2019:106), dichotomous approaches related to psychiatric nurses’ roles limit therapeutic potential as well as positive patient outcomes.

A literature review (Grabbe & Miller-Karas 2017:8; Hurley et al. 2022:Abstract; Ngooi et al. 2022:59; Rosendahl et al. 2021:56–67; Yoshinaga, Tanoue & Hayashi 2022:48) of group therapy models – facilitated by psychiatric nurses in the treatment of patients with mood disorders – revealed limited information in South Africa. The same applies globally. In some instances, literature revealed that psychiatric nurses incorporate group therapy for patients with mood disorders based on the cognitive behavioural therapy model; however, these also seemed limited. The cognitive behavioural approach is rooted in a psychoeducational approach. It uses cognitive restructuring as a means to address dysfunctional thought patterns that result in related dysfunctional emotional and behavioural responses among patients with mood disorders, especially patients with depressive disorders (Visagie 2017; Yoshinaga et al. 2022:48). Ngooi et al. (2022:59) explored activity-based group therapy’s value in increasing self-efficacy among patients with mental disorders. Their findings suggested that this kind of intervention (based on a behavioural activation model) is positively associated with patients’ behavioural coping, self-efficacy and mental well-being. However, this model was developed and implemented by occupational therapists in an acute inpatient setting.

According to Soltis-Jarrett (2020:364), the Society for Psychiatric Mental Health adopted constructs of Peeks’ (2013) lexicon for behavioural health integration. It deemed behavioural and substance use models as beneficial in treating patients with mental disorders. Grabbe and Miller-Karas (2017:4, 5, 8) also determined that a trauma-based resilience model showed positive outcomes in building resilience among patients who experienced trauma, which can be used in group or individual format. According to these authors, this model proposes the implementation of nine skills, with the first six skills referred to as the community resilience model. The latter focusses on preventative mental wellness and self-care and can be implemented for emotional stabilisation during times of emotional distress. These skills include tracking, resourcing and resource intensification, grounding, gesturing, help now and shift and stay survival responses. This model seems to embrace the nursing ethos and provides a new pathway for mental health care, but the core focus is trauma and not mood disorders.

The reviewed literature indicates a clear knowledge gap pertaining to a nursing model for the implementation of constructive group therapy for patients with mood disorders. To address this gap, a model for psychiatric nurses to facilitate constructive group therapy for patients with mood disorders was developed, described and evaluated.

### Description of the South African model for the facilitation of constructive group therapy

The model was developed as a frame of reference for psychiatric nurses to facilitate constructive group therapy for patients with mood disorders (Visagie 2023:97). The model comprised three phases: the relationship phase, the working phase and the termination phase (Visagie 2023:99). In the relationship phase, the focus was on initiating and promoting dynamic interaction between the psychiatric nurses facilitating constructive group therapy for patients with mood disorders and among the patients themselves and to provide a positive learning environment. Dynamic interaction between the psychiatric nurses and patients with mood disorders, and among the patients themselves, was vested in the psychiatric nurses’ ability to establish a therapeutic relationship with patients attending constructive group therapy sessions. The therapeutic relationship was built on the premise of trust by portraying a warm and caring attitude towards the patients with mood disorders attending the group sessions. Trust is an essential ingredient in building a therapeutic relationship (Girard et al. 2021:2; Moreno-Poyat et al. 2017:2; Muhorakeye & Biracyaza 2021:4–5).

The psychiatric nurses furthermore had to create a positive learning environment by establishing an atmosphere of non-judgement, respect, safety and acceptance using therapeutic communication skills during the facilitation of group sessions. The positive learning environment ensured interactive engagement between the psychiatric nurses and the patients with mood disorders, as well as among the patients themselves. During the relationship phase, the psychiatric nurses had to promote open communication, allowing the patients to express their feelings, perceptions, experiences and concerns without judgement. Promoting free expression without being judged instiled self-worth and a sense of value among the patients who attended the constructive group therapy sessions that psychiatric nurses facilitated. According to Loose (2020:n.p.), learning can only take place through interactive engagement, vested in a climate of respect. Once these activities were established, progress to the working phase followed.

In the working phase, the model focussed on how psychiatric nurses mobilised resources and enabled interactive engagement between themselves and the patients, as well as among the patients themselves. The aim was to promote therapeutic factors by facilitating constructive group therapy for patients with mood disorders (Visagie et al. 2023:127). According to the Theory of Health Promotion in Nursing (THPN) (University of Johannesburg 2017:7), the mobilisation of resources entails both external and internal resources in the physical, social and spiritual domains in which the person interacts. Mobilising external resources entailed psychiatric nurses creating a safe therapeutic space within the unit and the group setting. Patients experiencing emotional distress often feel hopeless and helpless (Uys & Middleton 2020:327) and need to feel safe within the unit and the group. Internal resources encompassed psychiatric nurses’ self-awareness to build a therapeutic relationship and portray empathy related to patients’ feelings, perceptions and experiences. Empathy refers to the ability to authentically relate to the feelings and experiences of others (Kneisl & Trigoboff 2014:29). The safe therapeutic group space and effective communication skills promoted interactive engagement to explore and reflect on patients’ feelings, perceptions and related behavioural responses. According to McAllister et al. (2019:2) and Okun and Kantrowitz (2015:75–76), facilitative communication skills foster authentic interaction, setting the table for the actual work to be done during the working phase.

Promoting the therapeutic factors underpinning group therapy served as the vehicle driving personal change and therapeutic growth among patients with mood disorders. These factors include hope, universality, shared information, altruism, interpersonal learning, imitative behaviour, group cohesion, socialisation, ventilation, corrective recapitulation of the primary family group and existential factors (Wedding & Corsini 2014:613). Patients with mood disorders experience emotional turmoil. Therefore, creating a safe space to vent and drawing group members’ attention towards shared vulnerabilities, negative feelings and life experiences promoted universality and provided a sense of hope. The psychiatric nurses used the group space as a platform for sharing information, providing psychoeducation related to mood disorders, coping mechanisms and coping skills. Facilitating constructive group therapy served to create altruism, thereby creating meaning for the individual group members as well as the collective group. According to Johnson (2014:2), the creation of meaning refers to factors that make a person or group feel valued, making their lives purposeful.

Moreover, interactive engagement fostered interpersonal learning by allowing group members to reflect on their feelings, perceptions and behavioural responses without being judged. This promoted group cohesion and socialisation. The psychiatric nurses assisted the patients in exploring, analysing and applying experiential learning and making constructive adaptations related to earlier familial roles and conflicts. Creating a therapeutic alliance with the patients attending group sessions fostered self-realisation, enabling patients to take ownership and responsibility for their emotional experiences and related behavioural responses through therapeutic growth and personal change.

The working phase was followed by the termination phase. It served to provide the psychiatric nurses facilitating constructive group therapy for patients with mood disorders with the opportunity to ‘say goodbye’ to their patients. In the process, they also assessed patients’ ability to independently function and cope with life stressors outside the safety of the group space and the inpatient unit. During this phase, the psychiatric nurses provided the patients an opportunity to reflect on their experiences attending constructive group therapy facilitated by psychiatric nurses and to express their feelings related to its termination. They were also allowed to reflect on their journey of empowerment, therapeutic growth and personal change. This made the patients feel valued and provided a stepping stone towards discharge.

### Aim

This study described psychiatric nurses’ experiences in implementing a model to facilitate constructive group therapy for patients with mood disorders.

## Research methods and design

The study was conducted in two phases. Phase one entailed psychiatric nurses working within the institution where the study was conducted, in the Free State province, South Africa, receiving training on and then implementing the model to facilitate constructive group therapy for patients with mood disorders. Phase two involved a qualitative evaluation of the model’s implementation.

### Phase one

Training on the model was provided to the psychiatric nurses using a workshop with the aim of empowering them to implement the model. Because of staff shortages and work schedules, two half-day workshops were conducted. Follow-up sessions were held for clarification purposes and questions. Six participants participated in the workshop, but only four psychiatric nurses were able to implement the model over 3 months.

### Phase two

Phase two entailed an evaluation of the model’s implementation. The evaluation process occurred directly after the workshop, 1 month after implementation and again 3 months after implementation. A qualitative, exploratory, descriptive and contextual research design was used during this study.

### Setting

This study was conducted at a public psychiatric hospital’s inpatient unit for patients with mood disorders in South Africa’s Free State. The Free State is one of nine provinces in South Africa, with a population of 2 745 590 (Statistics South Africa 2023). Patients are referred from private and public clinics and hospitals by psychiatrists. The unit is a psychotherapeutic unit with a bed capacity of eight, where both male and female patients with mood disorders are admitted. Patients admitted to the unit receive individual therapy and attend occupational therapy and group therapy facilitated by psychiatric nurses. The ward programme encompasses a multidisciplinary team approach, and patients receive pharmacotherapy from a psychiatrist.

### Study population and sampling strategy

The population was psychiatric nurses working in an inpatient unit with patients with mood disorders. Participants were purposively sampled to participate in this study. Purposive sampling refers to selecting participants for inclusion in a research study based on characteristics of interest related to the phenomenon being investigated (Boswell & Cannon 2020:326; Holloway & Galvin 2017:144; Liamputtong 2022:276; Pope & Mays 2020:49). Six psychiatric nurses were purposively sampled and attended the workshop, but only four were involved in the model’s implementation over a period of 3 months.

The inclusion criteria were psychiatric nurses working in an inpatient unit with patients with mood disorders. Participants had to be able to implement the model to facilitate constructive group therapy for patients with mood disorders for 3 months. Participants had to have an adequate grasp of English or Afrikaans and were in the age range 36–58 years. Participants had an average of 11 years (range 5–15 years) of work experience in psychiatric nursing. All four participants had a diploma in nursing (general nursing, midwifery, community health nursing and psychiatric nursing) and were full-time employees working in an inpatient unit for patients with mood disorders.

### Data collection

Data were collected by conducting individual phenomenological interviews with the four participants. The interviews lasted approximately 30 min – 45 min. The interviews were conducted in English by an independent field worker directly after the workshops, 1 month after implementation and again 3 months after implementation. Interviews were conducted in the unit, which was convenient for the participants. Participants, who were interviewed directly after the workshop, were asked: ‘How did the model work for you?’ Interviews were then repeated one month after implementing the model, and the participants were asked: ‘How did the implementation of the model work for you?’ Interviews 3 months after the model’s implementation again enquired: ‘How did the implementation of the model work for you?’

The independent fieldworker who conducted the interviews used facilitative communication skills, including probing, clarification, reflection and summarising in order to fully explore the psychiatric nurses’ experiences of the model’s implementation. Field notes were taken during the interviews, and the interviews were audio-recorded, transcribed and analysed using thematic coding. A total of 14 interviews were conducted, and data saturation was reached after the interviews’ completion.

### Data analysis

Data analysis is used to interpret, theorise and make sense of collected data and to assign meaning to the data (Pope & Mays 2020:117). In this study, thematic coding was used to analyse the data. According to Creswell (2016:174), thematic coding entails organising and categorising raw data into smaller parts to identify relevant important themes and categories. This process is used to make sense of the data by naming and labelling the components thereof (Holloway & Galvin 2017:292). An independent coder with extensive knowledge and experience in qualitative research and a PhD in psychiatric nursing was selected to reduce bias (Grove, Gray & Burns 2015:89). A consensus discussion was held between the author and the independent coder regarding the results.

### Trustworthiness

The following criteria to ensure trustworthiness were implemented: credibility, transferability, dependability and confirmability (Polit & Beck 2021:1139). Credibility was ensured by prolonged engagement with the psychiatric nurses who implemented the model. An independent coder who is knowledgeable in the field was consulted on the findings, and a consensus on the research themes was reached. Transferability was ensured by describing the participants extensively and supporting findings with quotations. Dependability was promoted by having a dependability audit, where three supervisors evaluated the study before it was presented to external assessors. Confirmability was improved by keeping a dense record of the research process for other researchers to replicate the process and draw similar conclusions.

### Ethical considerations

Ethical clearance for this study was obtained from the University of Johannesburg’s Faculty of Health Sciences Research Ethics Committee (REC-703-2020), the institutional ethics committee and the Free State Department of Health. Permission to conduct the study was also obtained from the chief executive officer of the institution before data collection. Written and verbal consent was obtained from all participants. Four principles were considered and adhered to when conducting research, namely autonomy, non-maleficence, beneficence and justice. Participants were assured of their anonymity and confidentiality. Thus, the participants’ real names were not used in the study; instead, participants were referred to by a number. Participation in the study was voluntary, and withdrawal was welcomed with no penalties. Collected data were only accessible to authors and the independent coder.

## Results

Four themes emerged from the findings of this study. The psychiatric nurses experienced the model as a supportive tool to facilitate constructive interactions among patients with mood disorders. The psychiatric nurses also found the model beneficial as it created a safe space for patients with mood disorders to share and deal with their challenges. The model assisted the psychiatric nurses in facilitating patients’ optimal functioning outside the hospital setting. The psychiatric nurses also shared that the model assisted them in improving their skills on a personal and professional level (Table 1).

**TABLE 1:** Summary of themes.

Themes	Description
Theme 1	The psychiatric nurses experienced the model as a supportive tool to facilitate constructive interactions among patients with mood disorders.
Theme 2	The model created a safe space for the patients with mood disorders to share and deal with their challenges.
Theme 3	The model assisted the psychiatric nurses in facilitating patients’ optimal functioning outside the hospital setting.
Theme 4	The model assisted the psychiatric nurses in improving their personal and professional skills.

*Source*: Visagie, H.M.P., 2023, ‘A model to assist psychiatric nurses in facilitating constructive group therapy for patients with mood disorders’, PhD thesis, University of Johannesburg

### Theme 1: The psychiatric nurses experienced the model as a supportive tool to facilitate constructive interactions among patients with mood disorders

The psychiatric nurses experienced the model as a supportive tool to facilitate constructive interaction among patients with mood disorders. Participants shared that the model was beneficial, as it improved interaction between themselves and the patients, as well as among the patients themselves. The following quotes reflect how the psychiatric nurses experienced the model as a supportive tool that improved interaction among patients:

‘How they interact with each other, sometimes they will learn, they will interact and when they do, let them talk to each other.’ (P4, female, 52 years, a month after the workshop)

‘To get the experience to interact with each other.’ (P5, female, 36 years, directly after the workshop)

The psychiatric nurses viewed the model as a supportive tool as it assisted patients in supporting each other. The following quote supports this statement:

‘When they start to be close to one another they will start to be able to help each other and sometimes.’ (P2, female, 53 years, directly after the workshop)

Another participant said:

‘Where they support each other, even the facilitator asked them to support each other.’ (P4, female, 52 years, 3 months after the workshop)

The psychiatric nurses learned from the model, improving their ability to effectively facilitate constructive group therapy for patients with mood disorders. The following quotes demonstrate this aspect:

‘But, for me it was really a learning experience, how you get them to open up with each other. Now, I am also enabled to help them, also to give them information about the emotions, how to deal with it, also to, uh, there was no judgement.’ (P5, female, 36 years, a month after the workshop)

‘We were reaching our goal, the aim of group therapy.’ (P4, female, 52 years, a month after the workshop)

### Theme 2: The model created a safe space for the patients with mood disorders to share and deal with their challenges

Patients with mood disorders experience emotional distress. They feel vulnerable, alienated from others and their world and fear being judged. The psychiatric nurses experienced the model was beneficial in assisting them to create a safe space for patients to share their feelings and perceptions and to work on their challenges. The model’s implementation ultimately helped the patients to open up. The following direct quotes reflect the psychiatric nurses’ experiences of patients being able to open up during constructive group therapy:

‘They learn to open up, focus on, not focussing on me being in charge of the group … they were opening up. You will see them like interacting towards another person.’ (P4, female, 52 years, a month after the workshop)

‘Patients being disclosing, or being open with us the groups were, they were open. They were willing and they were open, and they participated.’ (P3, female, 58 years, a month after the workshop)

Participants mentioned the model was a supportive tool in teaching patients to trust the psychiatric nurses, as well as other members of the group. The following quotes reveal how the model taught patients to trust others:

‘When they open up, when they begin to trust, when they see they won’t be judged.’ (P1, female, 38 years, a month after the workshop)

‘They also give hope and so that she can be trusting, so that when she is trusting and have hope, she can open more.’ (P4, female, 52 years, directly after the workshop)

The psychiatric nurses viewed the model as beneficial as it taught patients to learn from one another. The following quotes support this statement:

‘It does help because they learn from each other, because they will see you are like that, this problem that is bigger than me, it is just different.’ (P4, female, 52 years, a month after the workshop)

‘They can learn a lot from each other, you know sometimes you think you’re problem are bigger than the others.’ (P5, female, 36 years, a month after the workshop)

### Theme 3: The model assisted the psychiatric nurses in facilitating patients’ optimal functioning outside the hospital setting

The psychiatric nurses perceived the model as beneficial as it assisted them in empowering patients with mood disorders to cope effectively with life’s stressors outside the hospital setting. The following quote supports this statement:

‘The group helped them to go and function better outside.’ (P3, female, 58 years, a month after the workshop)

The psychiatric nurses deemed the model as enabling them to ensure positive patient outcomes and prepare patients for discharge. The following quotes portray these aspects:

‘Yes, when the patients go out, at least, the patients can make decisions and see, can deal with stressors outside.’ (P4, female, 52 years, a month after the workshop)

‘They get help, when they go out, when we discharge them, they can face the world.’ (P5, female, 36 years, 3 months after the workshop)

Another participant reflected:

‘It is the step towards, uhm, obtaining the main objective of the group and for the patients to be able to cope outside.’ (P4, female, 52 years, a month after the workshop)

The psychiatric nurses perceived the model as assisting them to ensure better patient outcomes. The following quotes demonstrate this aspect:

‘You see the change in them, now you will see them smiling, being comfortable, talking about their own experiences.’ (P4, female, 52 years, 3 months after the workshop)

‘Really there is an improvement in the patients.’ (P5, female, 36 years, 3 months after the workshop)

### Theme 4: The model assisted the psychiatric nurses in improving their personal and professional skills

The psychiatric nurses experienced the model as a helpful tool that improved their skills on a professional and deeper personal level. The model empowered them to interact therapeutically with patients with mood disorders through the facilitation of constructive group therapy. The following quote supports this aspect:

‘It influenced me positively, it helped me thinking, thinking I would like to do this duty.’ (P2, female, 53 years, directly after the workshop)

One participant reflected on the following quote relating to personal change:

‘So, it helped me a lot personally. Sometimes you are down, so, you can also use those techniques on yourself.’ (P4, female, 52 years, 3 months after the workshop)

The psychiatric nurses experienced the model as a tool that empowered and equipped them with a sound knowledge base pertaining to therapeutic interaction with patients with mood disorders. It also improved their competency in facilitating constructive group therapy for patients with mood disorders. They realised that they could make a meaningful and valuable contribution to patient care and outcomes; this enhanced self-actualisation. The following quote portrays these aspects:

‘Uh, it is almost like almost I feel important. I do feel important to my patients, important in a way, uh, and to myself, I do have self-esteem, unlike previously.’ (P3, female, 58 years, 3 months after the workshop)

The psychiatric nurses experienced the model’s implementation as a platform for boosting confidence and promoting job satisfaction. The following quotes highlight the psychiatric nurses’ experiences of improved self-esteem and job satisfaction:

‘The implementation worked very well for me. Even, now, I, I feel confident, having confidence to talk to the patients.’ (P5, female, 36 years, 3 months after the workshop)

‘Am confident with it. Hmm, amongst other things, I feel good about myself … even myself I do have self-esteem unlike previously.’ (P3, female, 58 years, 3 months after the workshop)

Another participant said the following quote related to patient outcomes and job satisfaction:

‘You, as a sister in the ward, also as matron in the ward, you will see the progress in your patients, especially those suffering from depression and what, and you see their mood is uplifting, you feel happy as a person, you feel happy about that.’ (P4, female, 52 years, 3 months after the workshop)

The psychiatric nurses further reflected that they experienced the model as helpful in improving communication skills. Improved facilitative communication skills strengthened the therapeutic nurse–patient relationship and therapeutic interaction with patients with mood disorders. The following quotes support this aspect:

‘Model helped us to be more of a listener. So, the model taught me to be more of a good listener.’ (P1, female, 38 years, a month after the workshop)

‘Even myself I have to learn to talk or face my problems.’ (P2, female, 53 years, directly after the workshop)

The psychiatric nurses said the model and its implementation enhanced therapeutic growth and personal change through a journey of introspection and self-discovery, creating self-awareness:

‘I was able to realise myself that is, to know where I lack, hmm. To know each other’s problems, maybe sitting-there thinking that you are the one whose undergoing bad things in your life and then, and then when you realise that you are not the only one, you feel relaxed.’ (P3, female, 58 years, a month after the workshop)

‘I should say, think I also learned that before I go and present the group, I should also know myself in totality.’ (P2, female, 53 years, directly after the workshop)

## Discussion

Psychiatric nurses are key players in providing mental health care to patients with mood disorders. The four themes identified from this study and participants’ related experiences facilitating constructive group therapy for patients with mood disorders illuminated the benefits for patients and psychiatric nurses who participated in the study. Psychiatric nurses experienced the model as a beneficial tool that assisted them in facilitating constructive group therapy for patients with mood disorders. According to literature (Chinn, Kramer & Sitzman 2022:168, 171; Polit & Beck 2021:179), nursing models are beneficial to clinical nursing practice. They provide valuable guidelines and structure for continuous improvements in clinical nursing practice, enhancing excellence in the field and ensuring positive patient outcomes. The purpose of nursing models is to resonate as a source of empirical knowledge, promote a clearer understanding of nursing practice and support and guide clinical nursing practice (Stevenson & Waite 2011:125; University of Johannesburg 2017:10).

In this study, the psychiatric nurses experienced the model as easy, and they understood its implementation. Some participants reflected that they could relate to the model, as the phases of its implementation were embraced by the nursing process with which they were familiar. Nursing models therefore provide structure and guidance in addressing challenges and priorities in nursing as a professional discipline embedded in empirical science by creating opportunities for discovery and innovation (Chezak et al. 2021:451; Rojas-Rivera, et al. 2022:6).

This model also embraced the importance of the therapeutic relationship between psychiatric nurses and patients with mood disorders attending constructive group therapy facilitated by psychiatric nurses. The therapeutic nurse–patient relationship is vested in trust and being valued. Nursing models align nursing practice with values, creating meaning in nursing and benefiting patients. Nursing models also allow nurses to become aware of their contribution to nursing practice and patient care (Stalling-Weldon, Hajewski & Shirey 2016:4). The participants in this study confirmed that the model served as a valuable source of knowledge, promoting experiential learning and interaction with patients with mood disorders.

The participants experienced the model as a supportive tool to facilitate constructive interaction between psychiatric nurses and patients with mood disorders, as well as among the patients themselves. Constructive interaction in the safe space of the group setting enabled patients to tap into their inner resources and deal with challenges related to mood disorders. Humans are social creatures with a deep-seated need to engage, belong and be valued by others (Ray et al. 2019:260; Wolff 2019:64). Constructive interaction therefore makes patients feel safe, accepted and valued by psychiatric nurses and other group members. The psychiatric nurses observed that constructive interaction also assisted the patients to freely express their feelings, perceptions and experiences. Sharing and learning from each other fostered universality and group cohesion. Through interaction in the safe space of the group setting, patients learned to support each other. Being supported and not feeling alone anymore also provided symptom relief. Interpersonal learning allows patients with mood disorders to realise they have the power to make the necessary changes to manage their mental illness and promote mental health (Kneisl & Trigoboff 2014:615; Uys & Middleton 2022:236; Yalom & Leszcz 2005:xiii).

According to the psychiatric nurses, the model assisted them in facilitating constructive group therapy effectively. The psychiatric nurses became acutely aware of their therapeutic role in treating patients with mood disorders, specifically pertaining to their role in providing psychotherapeutic interventions – in this case, constructive group therapy for patients with mood disorders. According to literature, group therapy is an important treatment modality for patients with mood disorders. However, this type of treatment is rarely implemented by psychiatric nurses (Gaskell et al. 2022:54; Medina et al. 2022:372; Novick & Swartz 2019:245). Being able to facilitate constructive group therapy resulted in therapeutic growth and personal change among patients with mood disorders. Observing patients’ progress and improvements provided the psychiatric nurses with a sense of achievement. As a result, the psychiatric nurses felt they were of value to the patients, which empowered and motivated them. Healthcare providers’ empowerment and motivation are essential factors in creating positive patient outcomes (Saleh, Eshah & Rayan 2022:2).

The model provided a safe space for patients with mood disorders to share and deal with their challenges. According to the psychiatric nurses, they witnessed patients working on themselves, becoming their own vehicles of change. The psychiatric nurses verbalised that the group setting provided a safety net where patients with mood disorders could interact freely. The patients attending group sessions could open up and express their feelings, perceptions and experiences without being judged.

Attending group therapy for the first time can be anxiety provoking, and patients often fear being judged by other group members and the psychiatric nurses facilitating the group sessions (Visagie et al. 2020:8). The psychiatric nurses’ ability to create a sense of safety and acceptance in the group setting was thus vested in the therapeutic alliance between them and the patients with mood disorders attending group sessions. Creating a therapeutic alliance built on trust ensured that the psychiatric nurses and patients worked together to achieve therapeutic growth and personal change through constructive group therapy. Berman (2019:190) states that trust is essential to help patients feel safe in the therapeutic inpatient unit and the group setting. Creating a sense of being safe and valued within the inpatient unit and group setting is critical in psychiatric nursing. Feeling safe and secure enhances free expression, interpersonal learning, autonomy, self-reflection and self-esteem (Bermam 2019:450; Pelto-Piri et al. 2019:8). Feeling safe and interacting with psychiatric nurses and other group members ultimately fostered patients’ experiential learning. According to Fitzpattick and McCarthy (2016:49), experiential learning is holistic in nature and entails more than simply a cognitive, affective or behavioural process. Authentic learning requires perceptive, cognitive, affective and behavioural processes and responses to be merged with life experiences.

During the model’s implementation, the psychiatric nurses witnessed patients improving their optimum level of functioning even outside the hospital setting. Patients were allowed to share their feelings and perceptions about their improvement as a result of participating in group therapy during the termination phase and while being discharged from the unit. These aspects assisted the psychiatric nurses to ensure positive patient outcomes and prepare patients for discharge. According to Uys and Middleton (2020:236), patients might experience their discharge as stressful and perceive discharge as a loss. Discharge planning therefore forms an integral part of inpatient treatment for patients with mood disorders, so they are prepared for discharge and the termination of the therapeutic nurse–patient relationship.

Being able to reflect on one’s own improvement and feelings about being discharged enhances resilience and provides a means to cope with life stressors after discharge. Coping entails the ability to employ effort, deal with existential crises and emotionally taxing issues and overcome obstacles hampering self-actualisation (Johnson 2014:374). According to Hagen, Knizek and Hjelmeland (2017:33), group therapy should incorporate positive attributes related to life experiences, symptom relief and the potential to experience personal change and growth, aimed at establishing self-fulfilment and fostering hope. The psychiatric nurses witnessed patients’ therapeutic growth and personal change. Attending constructive group therapy enabled the patients to ameliorate their distress and explore and experiment with alternative behavioural responses in the safety of the group therapy space and within the therapeutic environment of the inpatient unit. This resulted in positive patient outcomes and experiences of inpatient treatment.

The psychiatric nurses experienced the model’s implementation as beneficial, as it equipped them with knowledge and skills on a professional and deeper personal level. Implementing constructive group therapy for patients with mood disorders empowered the psychiatric nurses with knowledge and skills. According to Chinn et al. (2022:272), empowerment refers to a person’s ability to grow and develop on a cognitive level, developing their full potential on a professional and personal basis. It also implies the ability to adapt to changes in the environment in which the person is functioning, thereby promoting the ability to overcome barriers hampering the individual’s level of functioning. By implementing constructive group therapy, the psychiatric nurses’ sense of self changed. The model’s implementation opened the door to a journey of self-discovery and enhancing self-awareness, and they viewed themselves as being competent in facilitating constructive group therapy for patients with mood disorders.

Being able to facilitate constructive group therapy for patients with mood disorders and witnessing how patients improved boosted their confidence and enhanced their self-worth and self-esteem. According to Johnson (2014:338), self-esteem entails a person’s evaluation of their self-worth. Thus, self-esteem is enhanced by changing the way you think about yourself. Facilitating constructive group therapy for patients with mood disorders provided psychiatric nurses with a platform to interact on a deeper therapeutic level with their patients through a journey of introspection, creating self-awareness. Positive feedback from patients made them feel good about themselves and their role in treating these patients, and this provided them with job satisfaction. Job satisfaction is described in the literature as a person’s emotional and cognitive perceptions and experiences of their occupational role, providing a sense of pleasure and achievement (Adamy etal. 2017:331; Rosana & Sopiah 2022:186; Singh & Onahring 2019:9–10).

To engage with patients with mood disorders on a therapeutic level, psychiatric nurses had to build a sound therapeutic nurse–patient relationship with them. Building a trusting relationship is key to all therapeutic interactions with patients with mental disorders (Halter 2022:n.p.; Kornhaber et al. 2016:544). Moreover, during the facilitation of constructive group therapy for patients with mood disorders, the psychiatric nurses had to portray empathy towards patients using facilitative communication skills. Being able to portray empathy requires psychiatric nurses to genuinely relate to patients’ feelings, perceptions and experiences, as well as related behavioural responses. According to Dickins et al. (2019:17), the ability to reflect empathy refers to a caring attitude being displayed towards patients, where patients feel accepted without judgement. Portraying empathy towards the patients attending the group session provided the psychiatric nurses with an opportunity to observe how instiling safety and trust promoted therapeutic growth and personal change among patients with mood disorders.

Valuable insights related to the process of facilitating constructive group therapy for patients with mood disorders emanated from the model’s implementation. The psychiatric nurses experienced the model as easy. They felt they could relate to the different phases of the model and its implementation, as these phases were used in all psychiatric nursing interventions and nursing care processes. Regarding the relationship phase, the psychiatric nurses expressed that, at times, they had to build rapport in a very short time because of rapid patient turnover. Building a therapeutic relationship during the relationship phase provides patients with a safety net where they can feel safe and accepted without being judged (Uys & Middleton 2020:166). This aspect sets the table for the therapeutic healing process. Using the therapeutic self in creating an authentic therapeutic alliance ensures positive outcomes (Halter 2022:n.p.).

During the working phase, the rapid patient turnover also resulted in patients joining group therapy at different phases, and some of the psychiatric nurses were concerned about patients not attending all sessions; for example, being discharged prior to the termination phase or being unable to complete the actual therapeutic work required during the working phase. In addition, the patients with mood disorders who attended the group sessions also disclosed to the psychiatric nurses that they wanted to be part of the whole process and were even prepared to attend sessions after being discharged. The working phase focussed on the therapeutic factors inherent to group therapy and included hope, universality, information sharing, altruism, interpersonal learning, imitative behaviour, group cohesion, socialisation, ventilation, corrective recapitulation of the primary family group and existential factors (Kneisl & Trigoboff 2014:615; Yalom & Leszcz 2005:xiii). The psychiatric nurses witnessed the interplay of these therapeutic factors while facilitating constructive group therapy sessions and became aware of how these factors promoted therapeutic growth and personal change. They witnessed patients’ improvement after being able to vent and becoming hopeful again, not feeling alone anymore. Being able to vent their feelings within the safety of the group space helped patients experience symptom relief and fostered a sense of hope. Instiling hope in patients with mood disorders is an important therapeutic factor, which has a positive impact on relieving emotional distress (Chang 2017:459). Participants observed that patients’ socialising skills improved, and they learned to help each other. Being able to help each other creates meaning and a sense of being of value to each other (Jonhson 2014:2). The psychiatric nurses felt that the working phase provided a safe platform to engage in experiential learning and embrace the opportunity to reflect on past and present familial roles.

The psychiatric nurses experienced the termination phase as the final stepping stone before discharge. They perceived this phase as essential in discharge planning and preparing patients with mood disorders to cope outside the group and hospital setting. Coping refers to the ability to employ effort, deal with existential crises and emotionally taxing issues and demands and overcome obstacles hampering self-actualisation, reducing the person’s resources (Johnson 2014:374). Kneisl and Trigoboff (2014:50–51) align themselves with this aspect by reflecting that the termination phase represents a crucial moment in the facilitation process.

In summary, the participants reflected on the important aspects of the model’s implementation to facilitate constructive group therapy for patients with mood disorders. Reflecting on the challenges they experienced during the implementation process gave the researcher valuable insight and recommendations for clinical psychiatric nursing practice, education and research.

### Strengths and limitations

Model implementation during this study was challenging because of personnel shortages, as the model was implemented during the coronavirus disease 2019 (COVID-19) pandemic. Therefore, only four participants participated in the model’s implementation. The unit where the study was conducted also has a rapid patient turnover, which resulted in patients being incorporated into group sessions during different phases. The psychiatric nurses had to build a trusting nurse–patient relationship in a very short time. Some patients were also discharged before the termination phase. Using in-depth phenomenological interviewing was a strength of this study, as the psychiatric nurses’ experiences could be examined in detail and depth.

### Recommendations

The following recommendations for clinical nursing practice, nursing education and future nursing research were derived from this study. The developed model has extensively and holistically contributed to the therapeutic growth, personal change and optimal functioning of patients with mood disorders and therefore has the potential to ensure positive patient outcomes in various contexts. This model could also be utilised in different clinical settings by different professional disciplines. Simultaneously, this model has the potential to empower psychiatric nurses on a personal and professional level. The model can benefit nursing education by providing a platform for examining sound nurse–patient relationships, therapeutic skills and competency in the provisioning of therapeutic interventions for patients with mental disorders. The model can be incorporated into undergraduate and postgraduate studies for psychiatric nursing students. Incorporating the model in nursing training provides an additional opportunity for further research about the value of the model in nursing education. Currently, there is no evidence for a model of this nature. The researcher therefore deemed it necessary for other researchers to conduct similar qualitative studies. The researcher also deduced from the study’s findings that the model could be implemented with any other group of patients who need to acquire therapeutic growth and personal change.

## Conclusion

The model’s implementation and evaluation provided a scientific platform enhancing the body of knowledge of clinical psychiatric nursing practice. The researcher also witnessed the effectiveness of the model’s implementation first-hand. The model’s implementation illustrated psychiatric nurses’ importance in treating patients with mood disorders and ensuring positive patient experiences and outcomes. The model’s implementation and evaluation also shed light on psychiatric nurses’ empowerment through a journey of self-discovery and awareness related to personal and professional growth.
